# Associations between perpetrator characteristics of child maltreatment, age of onset, and physical and mental health

**DOI:** 10.1186/s13034-026-01049-w

**Published:** 2026-02-17

**Authors:** Marie Fiedler, Danielle Otten, Andreas Jud, Elmar Brähler, Jörg M. Fegert, Vera Clemens

**Affiliations:** 1 Department of Child and Adolescent Psychiatry/Psychotherapy, University Hospital Ulm, Steinhövelstr.5, 89077 Ulm, Germany; 2 Department of Psychosomatic Medicine and Psychotherapy, University Medical Center of Johannes Gutenberg University of Mainz, Mainz, Germany; 3 Department of Psychosomatic Medicine and Psychotherapy, Leipzig University Medical Center, Leipzig, Germany; 4 German Center for Mental Health (DZPG), Partner Site Ulm, Ulm, Germany

**Keywords:** Child maltreatment, Perpetrator, Onset, Physical health, Mental health

## Abstract

**Objective:**

Child maltreatment (CM) is a major health risk factor. However, perpetrator characteristics have been largely understudied in representative samples. Our aim is to describe perpetrator characteristics of CM and to identify risk constellation connecting onset of CM and perpetrator relations. Moreover, we look at differential effects of perpetrator relations on long term physical and mental health.

**Methods:**

In a cross-sectional observational approach, a representative sample of the German population (*N* = 2515) was assessed regarding CM characteristics (ICAST-R), physical and mental health as well as sociodemographic information. We used social network analysis to describe co-occurrences of perpetrator groups and $\:{{\upchi\:}}^{2}$ tests to test differences in relative frequencies of perpetrator relations in onset groups. Multivariate Analysis of Variance and follow-up analyses were used to identify differences in sociodemographic, CM characteristics as well as health outcomes between onset groups. A Poisson regression was calculated to scrutinize the effect of perpetrator relation on physical and mental health.

**Results:**

Perpetrator multiplicity and interconnectedness of perpetrator groups were high. Different frequencies of perpetrator groups across age of onset were observed for overall CM and CM subtypes. Participants who reported adolescent CM onset were younger than those with earlier CM onset. CM perpetrated by parents and by professionals in child related environments increased the risk of physical multimorbidity in adulthood. CM perpetrated by parents, relatives and others in the household, and peers and partners increased the risk of psychiatric multimorbidity in adulthood.

**Conclusion:**

This study provides insights into the risk constellations of CM by examining perpetrator relation, multiplicity, and co-occurrence. We found group differences in onset as well as differential risks for physical and psychiatric multimorbidity by perpetrator relation. These findings highlight the need for future research and prevention strategies that more explicitly address perpetrator characteristics.

**Supplementary Information:**

The online version contains supplementary material available at 10.1186/s13034-026-01049-w.

## Introduction

Child maltreatment (CM), defined as “all types of physical and/or emotional ill-treatment, sexual abuse, neglect, negligence and commercial or other exploitation, which results in actual or potential harm to the child’s health, survival, development or dignity in the context of a relationship of responsibility, trust or power” [1], is a worldwide major health risk. Meta-analytic worldwide prevalence rates reach from 12.7% for sexual abuse up to 36.3% for emotional abuse [2], mostly comparable with prevalence rates in the German population [3, 4].

Meta-analytic research has shown a strong association between CM experiences and increased physical and mental health risk in adulthood [5–9].

Most of the conducted studies assessed CM with the Adverse Childhood Experience Checklist (ACE; [10] or Childhood Trauma Questionnaire (CTQ) [11] and used subtype multiplicity (number of experienced CM subtypes) or overall sum scores. In the last decade instruments for the assessment of CM like the Maltreatment and Abuse Chronology of Exposure (MACE; [12] and the Child Abuse Screening Tools Retrospective version (ICAST-R [13, 14], allow us to better capture victimization across contexts, perpetrator groups, severity levels, and chronicity as the recurrence of victimization over time [3, 15, 16].

Duration of CM and specific exposure times seem to have a big impact on physical health [17] as well as psychopathology [15, 18, 19]. Also, earlier onset of CM has been associated with more symptoms of anxiety and depression in adulthood [20] and increases the risk for repeated and chronic CM [21].

The impact of perpetrator characteristics on CM victims’ health outcomes is an understudied topic. As Bennett et al. [22] state, the interpersonal nature of abuse suggests that perpetrators are a key element to study. Studies on perpetrators of CM have been mostly limited to case reports from official registries or focused on specific perpetrator groups within certain subtypes, particularly sexual abuse [23–25] as well as high risk populations [26].

Recently, Bennett et al. [22] compared prevalence of perpetrators in emotional, physical and sexual abuse within one population of foster children and adolescents in the U.S (*N* = 503). They showed biological caregivers as the most reported perpetrators for physical and emotional abuse. However, adolescents also reported high levels of victimization from peers. For sexual abuse non-related adults were the most identified perpetrators overall, though adolescents showed the highest levels of victimization perpetrated by peers.

First representative data on prevalence of perpetrators of CM in Germany showed parents to be the most frequent perpetrators for neglect as well as physical and emotional abuse [27]. However, in a more recent assessment peers and partners were among the most frequent perpetrators for physical and emotional abuse, followed by parents and then siblings [28]. For sexual abuse neighbors were most frequent, followed by other relatives and peers [27], comparable with prevalence rates of Fiedler et al. [28] showing 10 times higher prevalence rates for sexual abuse perpetrated by persons outside the household than for those within.

High CM subtype multiplicity has been associated with impaired health [6, 29, 30]. Likewise perpetrator multiplicity (number of perpetrator groups through which CM was experienced) might be an important factor to study. Bennett et al. [22] looked at perpetrator multiplicity in a foster care sample. They found older youth reporting higher perpetrator multiplicity than younger groups and girls reporting more perpetrators of psychological and sexual abuse compared to boys. They also showed positive associations of perpetrator multiplicity with abuse severity, and chronicity.

More research is needed to clarify perpetrator characteristics of CM in terms of epidemiology, including perpetrator–victim relations, perpetrator multiplicity, and co-occurrence (i.e., the joint reporting of multiple perpetrator groups by the same victim). Examining these characteristics in the context of already established CM indicators, such as subtype multiplicity and timing variables, is essential for a more comprehensive understanding. The role of onset in relation to different perpetrator groups is important to clarify patterns and to identify potential risk constellations. Our study aims to address these gaps and to provide a more nuanced understanding of the associations between perpetrator groups and impaired health.

## Research questions

We focus on perpetrator characteristics of CM by describing them in a nationally representative sample, connecting it with age of onset as well as examining their differential effects on health. More specifically this study poses the following questions: (1) How many perpetrator groups are reported by individuals experiencing CM, and which perpetrator groups most frequently co-occur? (2) Do the distributions of perpetrator groups differ by age of onset (early childhood, middle childhood, and adolescence)? (3) Do individuals with CM onset in early childhood, middle childhood, or adolescence differ in perpetrator multiplicity, duration of CM and long-term physical and mental health? (4) How are different perpetrator groups associated with long-term physical and mental health?

## Methods

A representative sample of the German population from the age of 16 onwards was obtained by an opinion research institute based in Berlin, Germany (USUMA) between July and October 2021. First a systematic area sampling (ADM F2F Sampling Frame), based on the municipal classification of the Federal Republic of Germany, covering the inhabited territory of Germany, was used. Then private households were systematically selected using a random route procedure. The kish-grid method was used to ensure random participation. Participants had to have sufficient German language skills. They were given information about the research background and the procedure and provided informed consent. An interview was used to assess socio-demographic information. Afterwards they fulfilled the questionnaire section, with a researcher nearby to answer possible questions. The study was conducted in accordance with the Declaration of Helsinki and was approved by the Ethics Committee of the Medical Department of the University of Leipzig.

Overall, 5908 target persons were identified (response rate of 42.6%). The main reasons for non-participation were refusal of the selected household or targeted person and failure to contact persons in the household after four attempts. The final sample size existed of *N* = 2,515 individuals from 16 years onwards and is representative for the German population. The mean age was M = 50.1 (SD = 18.0) and the mean monthly net household income was M = 2782 euros (SD = 1603). 51.6% of participants are female, < 0.01% diverse. Due to the low frequency of diverse persons, this group (*N* = 1) was excluded from gender specific data analysis. 56.7% were employed (full- or part-time), 26.6% were retired, 6.9% were in education or vocational training, and 9.8% were not in regular employment (including unemployment, unpaid domestic and caregiving work, and marginal employment). Overall, 41.6% were married, 26.6% were divorced or widowed, and 31.7% were single.

### Measures

CM was assessed using the German version of the ISPCAN Child Abuse Screening Tools Retrospective version questionnaire (ICAST-R) [13, 14]. This is a retrospective screening tool designed for adults capturing experiences of neglect, as well as physical, emotional, and sexual abuse before the age of 18. For individuals reporting CM, follow-up questions addressed the age at the time of occurrence, frequency, and severity of the experiences as well as perpetrators. The validity of the ICAST-R has been demonstrated in a national German sample, showing adequate to good internal consistency based on McDonald’s Omega [27].

For each subtype of CM (neglect, physical-, emotional-, and sexual abuse) participants could confirm or affirm exposure to 5 acts or refrain from answering. Affirmation of exposure to at least one act was coded as having experienced the corresponding subtype of CM. For subtype multiplicity the different subtypes of CM were summed up leading to a score ranging from 0 to 4.

For each act of CM participants were asked to indicate the perpetrators either choosing from a list of proposed perpetrators (parents, siblings, teacher, neighbor, other adult, somebody in my home, instructor/supervisor/coworker) or specify them themselves. We then conducted a qualitative analysis using thematic reduction to consolidate the data. First, we examined all open-text responses to determine fit with the preset categories. Items not aligning were grouped into newly formed categories, following an inductive coding step, with iterative updates to the coding scheme. The resulting categories were iteratively reduced and consolidated until six new categories remained: (1) parents, (2) siblings, (3) relatives and others in the household (incl. partners of parents), (4) professionals in child-related environments (e.g. teachers, priests, doctors), (5) other adults (e.g. neighbors, strangers), and (6) peers and romantic partners. Based on the definitional framework of neglect as the failure of a caregiver to provide for a child’s basic needs, peers and partners as well as other adults were excluded as perpetrator group for this subtype [31]. For perpetrator multiplicity the different perpetrator groups of CM were summed up leading to a score ranging from 0 to 6.

Onset of CM was defined as the first time point participants experienced any subtype of CM. Due to possible recall bias [32], onset until the age of 3 (*N* = 38) was not considered. Specifically, individuals who also reported having experienced CM at the age of 4 were included in this age onset group (*n* = 35), the others (*n* = 3) were excluded for this study. Onset of CM thus ranged from 4 to 18. Three age groups of onset were created: 4–6, 7–12 and 13–18 years, based on developmental stages [33]. Duration was defined as the total number of years of experienced CM and calculated by summing up the time points leading to a score ranging from 0 to 18.

For physical health a physical multimorbidity score was calculated. Therefore, participants were asked if they suffered from one of the following seven diseases: obesity, diabetes mellitus, cancer, hypertension, myocardial infarction, chronic obstructive pulmonary disease (COPD), and incident of a stroke (binary answer format). A sum score of these self-reported diseases was built ranging from 0 to 7.

For mental health we calculated a psychiatric multimorbidity score ranging from 0 to 8. Participants were asked if they had ever received a diagnosis of a physician or psychotherapist for one of the following eight mental disorders: depression (incl. burn-out), anxiety, post-traumatic stress disorder (PTSD), personality disorder (borderline, others), eating disorder, attention-deficit/hyperactivity disorder (ADHD), schizophrenia and substance use disorder (alcohol or other drugs).

Sociodemographic data were assessed as follows: age in years and household net income in euros (answer categories were recorded continuously), and sex (dummy coded 0 for female, 1 for male).

### Statistical analyses

For statistical analyses SPSS version 29 and R Studio version 4.3.2 were used. Descriptive analyses were conducted for socio-demographic characteristics, physical and psychiatric multimorbidity as well as CM characteristics (duration, onset, subtype and perpetrators multiplicity).

A social network analysis was performed to describe co-occurrences of perpetrators. It allows visualization of multiple dimensions of perpetrator profiles. Node size describes number of connections, centrality in the network describes interconnectedness of a perpetrator, edge weights describe frequency at which two perpetrators were reported from one person [34].

Group comparisons were conducted by categorizing participants based on age of onset (4–6, 7–12, 13–18 years) and examining the relative frequencies of perpetrator relations depending on group membership. Three persons were excluded because of an age of onset before the age of 4. $\:{{\upchi\:}}^{2}$ tests were performed to test differences in relative frequencies of perpetrator relations in onset groups.

To examine the differences between the onset groups in household net income, age, duration of CM, perpetrator multiplicity as well as physical and psychiatric multimorbidity a Multivariate Analysis of Variance (MANOVA) was used. To identify specific differences between groups, follow-up univariate ANOVAs and post-hoc analyses were conducted. The Bonferroni correction and Tukey’s HSD test were applied to control for Type I error due to multiple comparisons. The effect sizes (η²) for each dependent variable were calculated to assess the magnitude of the differences between the groups. To account for potential imbalances in group sizes, the harmonic mean was applied to assess the homogeneity of variances across groups in the multivariate analysis.

Partial correlations of CM timing variables (onset, duration), subtype multiplicity and perpetrator characteristics were calculated. Furthermore, multiple regression analyses were calculated to look at the effect of perpetrator relations on physical and mental health. Since physical and psychiatric multimorbidity represented count data (ranging from 0 to 7 or 0 to 8) with independent observations, Poisson regression was applied. The results are presented in forest plots that compare incidence rate ratios (IRR). IRR shows for each one-unit increase in predictor X, the expected count of the outcome variable increases by XX%, holding all other variables constant. For interpretability, household net income was scaled by a factor of 100; all reported IRRs therefore refer to a 100 euro increase.

## Results

### Sample characteristics

In Table 1 the sample characteristics of CM and physical as well as psychiatric diseases are shown. Overall, 36.5% (*N* = 918) experienced at least one subytpe of CM, average onset of CM with 9 years and a mean duration of 5 years. Mean values of physical and psychiatric multimorbidity were M = 0.62 (SD = 0.99) and M = 0.31 (SD = 0.84), respectively. Diagnoses of at least one physical disease were reported by 928 (36.9%) and diagnoses of at least one psychiatric disease by 410 (16.3%) participants.

**Table 1:** Sample characteristics of study participants in N (%) /M ± SD

	Total	Female	Male
	2514	1297 (51.6)	1217 (48.4%)
Age	50.08 ± 18.04	50.60 ± 17.91	49.53 ± 18.17
Household net income	2782 ± 1603	2672 ± 1531	2898 ± 1670
Experienced any CM	918 (36.50)	474 (36.55)	444 (36.48)
Onset*	8.91 ± 4.10	9.13 ± 4.20	8.68 ± 3.97
Duration*	5.03 ± 3.95	4.89 ± 3.93	5.18 ± 3.96
Subtype multiplicity*	1.92±0.94	1.90±0.97	1.95±0.90
Perpetrator multiplicity*	1.91 ± 1.06	1.91 ± 1.06	1.91 ± 1.06
Physical multimorbidity	0.62±0.99	0.60±0.93	0.64 ± 1.04
Overweight	470 (18.7)	263 (20.3)	206 (16.9)
Diabetes Mellitus	218 (8.7)	103 (7.9)	114 (9.4)
Cancer	86 (3.4)	52 (4.0)	34 (2.8)
Hypertension	620 (24.7)	304 (23.4)	316 (26.0)
Myocardial infarction	82 (3.3)	26 (2.0)	56 (4.6)
Chronic obstructive pulmonary disease	45 (1.8)	20 (1.5)	25 (2.1)
Stroke	55 (2.2)	18 (1.4)	37 (3.0)
Psychiatric multimorbidity	0.31±0.84	0.38±0.97	0.24±0.68
Depression	294 (11.7)	188 (14.5)	106 (8.71)
Anxiety	149 (5.9)	109 (8.4)	40 (3.29)
PTSD	43 (1.7)	25 (1.9)	18 (1.48)
Personality disorder	52 (2.1)	34 (2.6)	18 (1.50)
Eating disorder	69 (2.7)	55 (4.2)	14 (1.15)
ADHD	21 (0.8)	8 (0.6)	13 (1.07)
Schizophrenia	4 (0.2)	1 (0.1)	3 (0.25)
Substance use disorder	110 (4.4)	27 (2.1)	83 (6.82)

CM = child maltreatment, * if any CM experienced

**Fig. 1:**
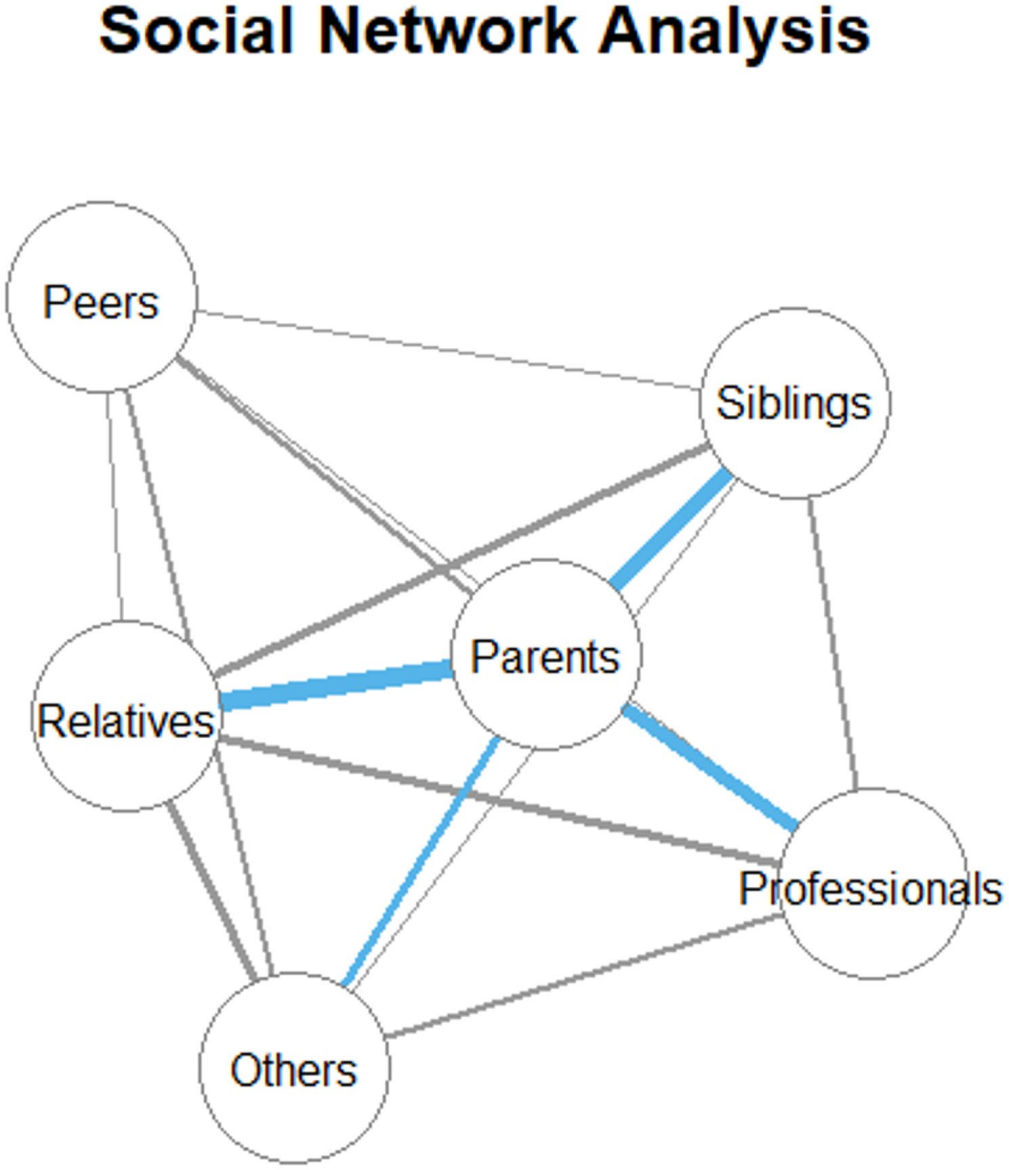
Social Network Analysis of CM perpetrator relations. Centrality describes higher interconnectedness of a perpetrator; edge thickness describes frequency at which two perpetrators were reported from one person (blue > 100; grey < 100). Some labels have been shortened to fit better within the network figure: Relatives and others in household, Professionals in child-related environments, Other adults and Peers and partners

Parents were the most frequent perpetrator group regarding neglect, emotional- and physical abuse. For sexual abuse other adults were the most frequent group (for a detailed overview see Table S1). The proportion of victims reporting multiple perpetrator groups was 53.8% overall and ranged from 28.9% for sexual abuse to 40.7% for emotional abuse (see Figure S1). Results of the social network analysis displayed in Fig. 1 show which perpetrator groups across all subtypes of CM often occur together. Parents were shown to be the most interconnected perpetrator group showing especially high co-occurrences with relatives and others in the household, siblings as well as professionals in child-related environments. Peers and parents are the least frequent co-occurred perpetrator group.

### Connecting perpetrator and onset of CM

**Fig. 2:**
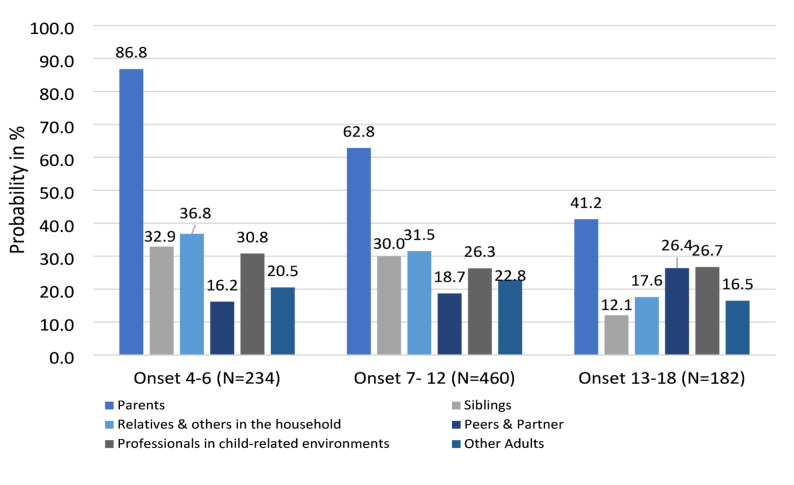
Relative frequencies of perpetrators categorized by onset

Onset of CM was split into three groups existing of 234 (26.7%) persons with CM onset between ages 4–6, 460 (52.5%) persons with CM onset between ages 7–12 and 182 (20.8%) persons with CM onset between ages 13–18 (see Fig. 2). $\:{{\upchi\:}}^{2}$-tests were conducted to test differences in relative frequencies of perpetrators in onset groups. Differences in relative frequencies of perpetrator groups in onset of overall CM were found in parents ($\:{{\upchi\:}}^{2}$ [2] = 94.54, *p* < .001**), siblings ($\:{{\upchi\:}}^{2}$ [2] = 26.74, *p* < .001*), relatives and others in household ($\:{{\upchi\:}}^{2}$ [2] = 18.94, *p* < .001**) as well as peers and partners ($\:{{\upchi\:}}^{2}$(2) = 7.21, *p* = .03*). While relative frequencies of parents, siblings and relatives and others in household decrease with later onset, parents remained the most frequent perpetrators in all three onset groups. Frequency of perpetration by peers and partners was highest in adolescence. Similar patterns were observed for physical abuse. For neglect, parents were the most frequently reported perpetrator group across all three age groups, with the relative frequency decreasing with age. Relatives and others in household were more frequently reported in middle childhood than in early childhood and adolescence. For emotional abuse the relative risk of being perpetrated by professionals in child related environments ranged from 27.1% to 28.3% across onset groups. For sexual abuse parents were the least frequent perpetrator group over all onset groups. For this subtype, relatives and others in household were the most frequent perpetrator group in early and middle childhood and peers and partners the most frequent perpetrator group in adolescence. For a detailed overview of relative frequency of perpetrators categorized by CM onset per subtype see Figure S2 and Table S3.

### Comparing perpetrator characteristics with other CM characteristics

The results of the MANOVA revealed a main effect for the onset groups [F(18, 6712) = 254.97, *p* < .001, Wilks’ Λ = 0.22, partial η² = 0.40]. In Table 2 descriptives of the onset groups as well as follow-up ANOVA tests and post-hoc Tukey HSD are shown. Post-hoc comparisons using Tukey’s HSD test revealed differences across all dependent variables.

Persons with early onset of CM had lower household net income than persons with no-CM (*p* < .05). Persons with an adolescent onset of CM were younger than those with an earlier onset of CM (*p* < .05). Groups differed in duration of CM and perpetrator multiplicity, with earlier onset being associated with longer duration and a higher number of perpetrators (both *p* < .05). The no-CM and adolescent-onset groups showed lower levels of physical multimorbidity than the early and middle childhood onset groups, which also differed from each other (*p* < .05). The no-CM group showed lower psychiatric multimorbidity than the CM groups. In addition, the early-onset group had higher levels of psychiatric multimorbidity than those with adolescent onset.

**Table 2:** Descriptives, univariate ANOVA and post hoc comparisons of onset groups

Dependent variable	No CM (M ± SD)	Onset 4–6 (M ± SD)	Onset 7–12 (M ± SD)	Onset 13–18 (M ± SD)	ANOVA, F(df)=, *p*, partial η²
Household net income	2892 ± 1633^a^	2541 ± 1761^b^	2614 ± 1499 ^ab^	2626 ± 1322 ^ab^	F(3,2348) = 6.22, *p*<.001, η² = 0.01
Age	49.70 ± 17.84 ^ab^	53.21 ± 17.19 ^a^	51.74 ± 18.96 ^a^	47.66 ± 18.20 ^b^	F(3,2348) = 4.41, *p*=.01, η² < 0.01
Duration of CM	- ^a^	7.61 ± 4.04 ^b^	4.41 ± 2.77 ^c^	2.04 ± 1.30 ^d^	F(3,2348) = 1654.74, *p*<.001, η² = 0.68
Perpetrator multiplicity	- ^a^	2.09 ± 1.20 ^b^	1.87 ± 1.07 ^c^	1.27±0.86 ^d^	F(3,2348) = 1497.16, *p*<.001, η² = 0.66
Physical multimorbidity	0.52±0.93 ^a^	1.04 ± 1.26 ^b^	0.78 ± 1.02 ^c^	0.54±0.86 ^a^	F(3,2348) = 21.64, *p*<.001, η² = 0.03
Psychiatric multimorbidity	0.14±0.52 ^a^	0.72 ± 1.17 ^b^	0.56 ± 1.10 ^bc^	0.42±0.96 ^c^	F(3,2348) = 59.00, *p*<.001, η² = 0.07

ANOVA p values Bonferroni-Corrected α = 0.0083; means within each row that do not share a common superscript letter differ significantly at *p* < .05, Tukey HSD post hoc test. Superscript letters are re-assigned for each outcome variable

Partial correlations of CM perpetrator characteristics with CM subtype multiplicity and duration were calculated. Pearson correlations of perpetrator multiplicity with CM subtype multiplicity (*r*=.85, *p*<.01) as well as duration (*r*=.74, *p*<.01) showed high positive associations. Perpetrator relations had a positive association with perpetrator multiplicity, subtype multiplicity and duration (highest for parents, followed by relatives and others in the household and siblings, for details see Table S4).

### Differential risk of perpetrator relation on long term physical and mental health

**Fig. 3:**
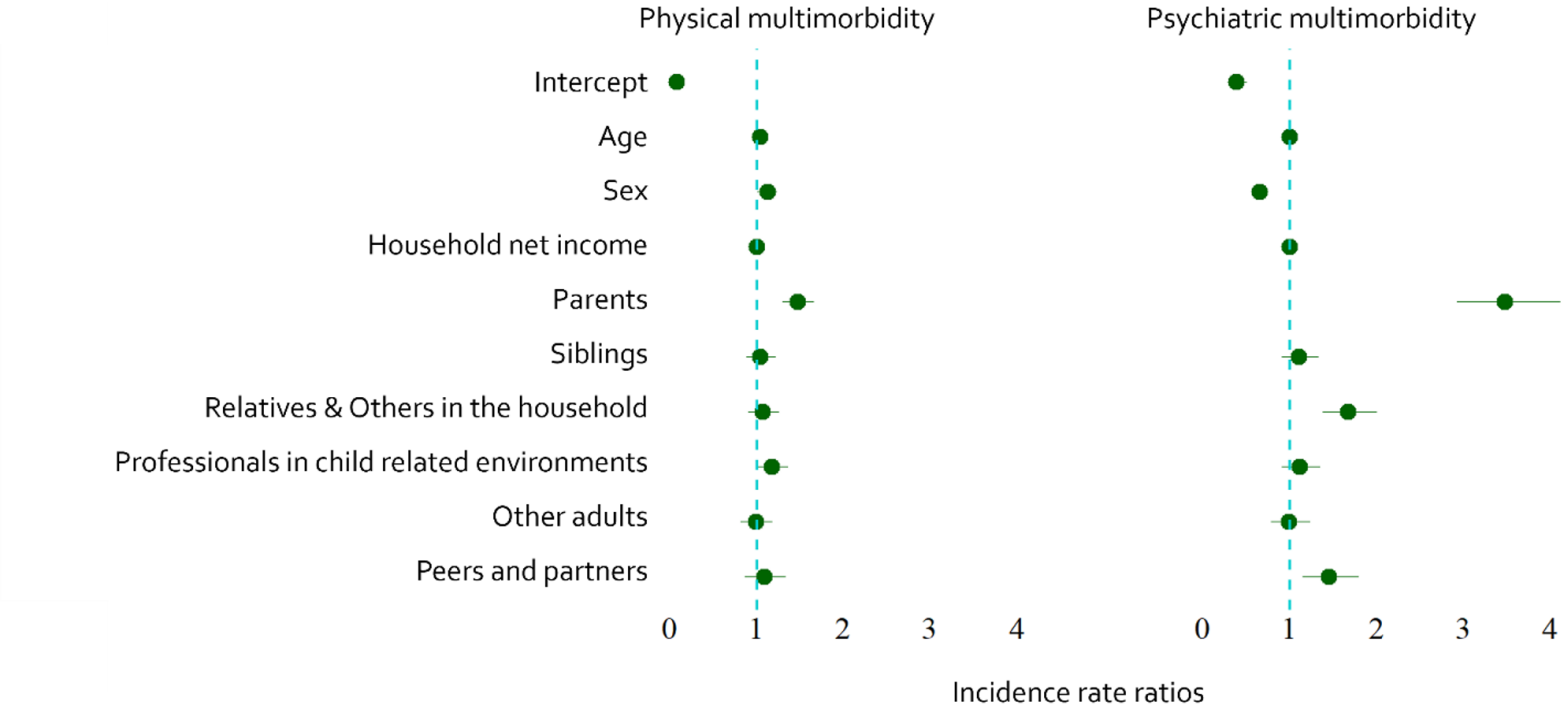
Forest plot of incidence rate ratios (IRR) with 95% confidence intervals for predictors of physical and psychiatric multimorbidity. Dots represent estimated IRRs, and horizontal lines show the 95% confidence intervals. The dashed vertical line marks the null value (IRR = 1) Household net income in 100 euros

A Poisson regression was conducted to examine whether perpetrator relation of CM increased the risk of long term impaired physical health while controlling for age, sex and household net income. The model was significant, χ² [9] = 905.4, *p* < .001, indicating that the predictors improved model fit compared to the null model. Moreover, the model was significant, χ² [6] = 70.38, *p* < .001, compared to the model with only control variables age, sex and household net income. In Fig. 3, IRRs for all predictors on physical health are shown. CM perpetrated by parents increased the risk of physical multimorbidity in adulthood by approximately 47% (IRR = 1.47, B = 0.36, SE = 0.06, z = 5.67, *p* < .001). CM perpetrated by professionals in child related environments increased the risk of physical multimorbidity by approximately 18% (IRR = 1.18, B = 0.16, SE = 0.08, z = 2.11, *p* = .04). Men had an increased risk of 13% of physical multimorbidity compared to women (IRR = 1.13, B = 0.12, SE = 0.05, z = 2.25, *p* = .02). The risk of physical multimorbidity increased by 4% (IRR = 1.04, B = 0.04, SE = 0.00, z = 23.24, *p* < .001) for each year increase in age. A higher household net income decreased the risk of physical multimorbidity (IRR = 0.99, B = 0.00, SE = − 0.01, z = -5.71, *p* < .001).

A second Poisson regression was conducted to examine whether perpetrator characteristics of CM increase the risk of long term mental ill health while controlling for age, sex, household net income. The model was significant, χ² [9] = 599.26, *p* < .001, indicating that the predictors improved model fit compared to the null model. Moreover, the model was significant, χ² [6] = 416.13, *p* < .001, compared to the model with only control variables age, sex and household net income. In Fig. 3 IRRs for all predictors on mental health are shown. CM perpetrated by parents increased the risk of psychiatric multimorbidity by 328% (IRR = 3.28, B = 1.19, SE = 0.09, z = 13.62, *p* < .001). CM perpetrated by relatives and others in the household increased the risk of psychiatric multimorbidity by 70% (IRR = 1.70, B = 0.53, SE = 0.09, z = 5.63, *p* < .001). CM perpetrated by peers and partners increased the risk of psychiatric multimorbidity by approximately 43% (IRR = 1.43, B = 0.36, SE = 0.11, z = 3.18, *p* = .001). Men had a 36% lower risk of psychiatric multimorbidity than women (IRR = 0.64, B = − 0.43, SE = 0.08, z = -5.62, *p* < .001). Each year increase in age decreased the risk of psychiatric multimorbidity by 1% (IRR= 0.99, B = − 0.01, SE = 0.00, z = -3.15, *p* < .001). A higher household net income reduced the risk of psychiatric multimorbidity (IRR = 0.99, B = − 0.01, SE = 0.00, z = -5.29, *p* < .001).

## Discussion

This study is the first to examine perpetrator characteristics of CM and their association with onset as well as long-term health outcomes in a representative sample. We addressed four main research questions concerning perpetrator groups of CM. First, we examined perpetrator multiplicity and the co-occurrence of perpetrator groups. Second, we investigated whether distributions of perpetrator groups differed by age of onset. Third, we investigated whether onset groups varied in perpetrator multiplicity and long-term mental and physical health. Fourth, we explored differences in long-term mental and physical health by perpetrator group.

Perpetrator–victim relations of our sample were described before [27]. Beyond that, we found perpetrator multiplicity with 53.8% of the persons with CM to have had multiple perpetrator groups notably high. The perpetrator groups are highly interconnected. Especially parents showed high co-occurrences with relatives and others in the household, siblings as well as professionals in child-related environments. Peers and parents were the less frequent co-occurring perpetrator group. This is in line with the research of Finkelhor et al. [35] showing high rates of CM recurrence and outlining pathways toward repeated exposure, emphasizing the role of dangerous communities and unsafe family environments as risk factors. Our results highlight that revictimization in CM not only occurs through the same perpetrators [36], but also across multiple perpetrator groups. This offers valuable insight into the distribution of perpetrator multiplicity. More research is needed on how individual, familial, and contextual factors jointly relate to perpetrator multiplicity and co-occurrence across settings, ideally using longitudinal designs to better understand temporal sequences and underlying mechanisms. Also, perpetrator multiplicity was highly positively associated with duration of CM as well as subtype multiplicity comparable with findings from Bennett et al. [22].

Relative frequencies of perpetrator groups differed across onset groups for overall CM as well as per subtypes, indicating specific risk constellations to have in mind for prevention of CM in early and middle childhood as well as adolescents. For overall CM we found relative frequencies of parents, siblings and relatives and others in household to decrease with later onset, parents however remained the most frequent perpetrators in all three onset groups. Frequency of perpetration by peers and partners was highest in adolescence. For emotional abuse professionals in child-related environments were consistently reported across age groups (27.1% in middle childhood; 28.3% in early childhood and adolescence) and were more prevalent than in other subtypes. This finding is in line with a systematic review reporting on high emotional abuse in school environments [37]. For sexual abuse parents were the least frequent perpetrator group over all onsets. Relatives and others in the household were the most frequent perpetrator group for early and middle childhood. Peers and partners were the most frequent perpetrator group in adolescence which is in line with Bennett et al. [22] research in a high risk population. These differential patterns demonstrate the importance of connecting onset and perpetrator characteristics to gain a deeper understanding of risk constellations.

The comparison of CM onset in different stages of childhood revealed differences in all dependent variables. Household net income was lower among individuals with CM onset in early or middle childhood, compared to those with a late onset or no history of CM. This association may reflect the role of low socioeconomic status as a risk factor for experiencing CM [38] as well as the reverse causality — early onset of CM contributing to lower socioeconomic status in adulthood [39]. The finding of lower current age in participants with no CM or CM onset in adolescence, compared to those with earlier CM onset, may suggest a trend toward a later onset of CM in more recent cohorts. While previous studies have shown stable age patterns by subtype (e.g., neglect in early childhood, sexual abuse in adolescence; [3, 40]), few have explicitly examined cohort-related differences in onset timing. Previous research has reported age-related differences in physical abuse, which have been discussed in relation to cohort-related changes in parenting practices [28, 41]. In Germany, the introduction of the legal right to a non-violent upbringing in 2001 and related information campaigns has been associated with a decline in physical punishment [42]. Which may help contextualize our finding of later CM onset among younger cohorts, however further research is needed to clarify these patterns.

As expected, the duration of CM was longer in individuals with earlier onset of CM. Additionally, perpetrator multiplicity increased with earlier onset. These findings imply that individuals who experienced CM at an earlier age not only endured it over a longer period of time but were also more likely to be exposed to multiple perpetrators. As psychotherapy research suggests children with history of CM have difficulties in sensing and setting boundaries possibly due to a lack of learning experiences of their own boundaries and acknowledgment of feelings as well as possible guilt and shame reactions, and therefore are at greater risk for revictimization [43–45]. Regarding the duration of exposure to CM, the close relationship between timing of onset and duration warrants further attention. The earlier the onset of CM, the longer the duration of exposure, which likely increases the risk of long-term harm. Therefore, it is crucial to consider risk constellation of onset periods with specific perpetrator groups to get better in prevention and intervention methods.

Individuals with late-onset or no history of CM had lower physical multimorbidity compared to those with CM onset in middle childhood. The highest physical multimorbidity was observed in individuals with early CM onset. Psychiatric multimorbidity was also higher in individuals exposed to CM in early and middle childhood, compared to those with onset in adolescence or no history of CM. These findings underscore the severe impact of CM on overall health, with earlier onset associated with higher levels of physical and psychiatric multimorbidity. This may reflect longer exposure periods and a higher likelihood of multiple perpetrator groups, as both indicators were also associated with earlier onset. Comparable with recent findings [17] showing a high importance of duration of CM for physical multimorbidity.

Furthermore, CM perpetrated by parents or by professionals in child-related environments increased the risk of physical multimorbidity in adulthood. This result is particularly important because both parents and teachers are among the adults with the greatest authority and influence in a child’s everyday life. Experiencing maltreatment in these relationships may especially undermine a child’s sense of safety and stability in fundamental social environments, which could contribute to long-term physiological stress responses that have been linked in prior studies to elevated risks for physical health problems [46, 47].

In terms of mental health, CM perpetrated not only by parents but also by other relatives or household members, as well as peers and partners, was associated with an increased risk for psychiatric multimorbidity. This suggests that psychological harm is not limited to abuse by primary caregivers but can also result from maltreatment in broader social contexts, including other close adults and within peers and partner situations. These social relationships, especially during adolescence, possibly play a central role in emotional development, and harmful experiences within them seem to have lasting effects on mental health comparable in severity to those caused by familial abuse.

### Strengths und limitations

A notable strength of this study is its large sample size (*N* > 2500), which is representative of the German population, ensuring good generalizability of the results. Additionally, the use of the ICAST is a key strength, as it provides detailed information on both the characteristics of perpetrators and the timing of CM. This stands in contrast to other commonly used instruments, which primarily measure subtype multiplicity but lack insight into the broader context of CM.

One limitation of this study might be the use of perpetrator groups such as there is no distinguishing between mothers and fathers. This lack of differentiation could potentially mask important nuances, as the role of CM by one parent compared to both primary caregivers may differentiate the long-term effects of CM. Additionally, the high associations among all CM characteristics present challenges in statistical comparisons, particularly in regression models, as they result in shared variance that can limit the ability to distinguish their individual effects. Future studies should consider alternative methods to assess the influence of these highly correlated variables. Also, we used retrospective self-assessment to collect data, which may involve recall bias and potential social desirability effects [48, 49]. Considering the substantial underreporting of CM, with global prevalence rates ranging from 0.3% to 0.4% in informant studies compared to 7.6% to 36.3% in self-report prevalence rates [2], which may result in severe selection bias, anonymous retrospective self-ratings continue to be a highly valuable source of information. Our study is observational and utilizes a cross-sectional design, thus no changes over time can be investigated nor can we draw causal inference from our work. The results may partly be driven by neglect as a subtype, particularly in terms of duration and co-occurrence, as parents are more likely to be the perpetrators of neglect. It may therefore be useful to consider neglect separately from other forms of CM since, by definition, neglect involves a more limited range of perpetrators, primarily caregivers [31]. This distinction could reveal different patterns and provide unique insights that may not be captured when all forms of maltreatment are considered together. Further research is needed to explore whether neglect presents a distinct pattern of effects on health outcomes compared to emotional, physical, and sexual abuse.

## Conclusion

Overall, our study contributes to a deeper understanding of the risk constellations associated with CM. By exploring perpetrator-victim relation, perpetrator multiplicity, and perpetrator co-occurrence, we gain valuable insights into the epidemiology of CM. Integrating these perspectives with the onset of CM further enhances our understanding of the complex risk factors at play. Moreover, our findings show high associations of CM perpetrator characteristics with long-term impaired health, underscoring the importance of incorporating perpetrator characteristics into future research on consequences of CM. From a clinical perspective, CM assessments should account for different perpetrator groups. In society we need greater awareness of perpetrator groups in conjunction with the onset of CM to inform more targeted prevention strategies. Furthermore our findings help to identify individuals at elevated risk for long-term mental and physical health problems after experiencing CM. Further efforts should focus on creating effective intervention strategies to mitigate the long-term effects of CM.

## Supplementary Information

Below is the link to the electronic supplementary material.

Supplementary Material 1.

## Data Availability

The datasets analysed during the current study are available from the corresponding author on reasonable request.
