# Supplementary material for: Associations between perpetrator characteristics of child maltreatment, age of onset, and physical and mental health
Source: Child Adolesc Psychiatry Ment Health. 2026 Feb 17;20:28. doi: 10.1186/s13034-026-01049-w (PMC12931050; doi:10.1186/s13034-026-01049-w)
Supplement: Supplementary file 1 — Supplementary Material 1. [file 13034_2026_1049_MOESM1_ESM.docx]

Supplementary Materials

| Table S1. Frequency of CM by perpetrators in N (%) in the whole sample | | | | | |  |
| --- | --- | --- | --- | --- | --- | --- |
|  | Neglect | Physical Abuse | Emotional Abuse | Sexual Abuse | Overall CM | |
| Parents | 242 (9.6%) | 357 (14.2%) | 356 (14.2%) | 19 (0.8%) | 574 (22.9%) | |
| Siblings | 103 (4.1%) | 100 (4.0%) | 129 (5.1%) | 19 (0.8%) | 242 (9.6%) | |
| Relatives and others in household | 92 (3.7%) | 94 (3.7%) | 146 (5.8%) | 71 (2.8%) | 265 (10.5%) | |
| Professionals in child-related environments | 47 (1.9%) | 53 (2.1%) | 160 (6.4%) | 27 (1.1%) | 234 (9.3%) | |
| Other adults |  | 58 (2.3%) | 71 (2.8%) | 82 (3.3%) | 184 (7.3%) | |
| Peers and partners |  | 60 (2.4%) | 81 (3.2%) | 73 (2.9%) | 174 (6.9%) | |

**Figure S1**. Perpetrator multiplicity overall and per subytpe of CM in %.

| **Table S2. Frequencies of co-occured perpetrator groups** | | | | | | |
| --- | --- | --- | --- | --- | --- | --- |
|  | 1. | 2. | 3. | 4. | 5. | 6. |
| 1.Parents | 0 | 181 | 191 | 143 | 102 | 70 |
| 2.Siblings |  | 0 | 96 | 74 | 51 | 42 |
| 3.Relatives and others in household |  |  | 0 | 86 | 81 | 39 |
| 4.Professionals in child-related environments |  |  |  | 0 | 67 | 45 |
| 5.Other adults |  |  |  |  | 0 | 54 |
| 6. Peers and partners |  |  |  |  |  | 0 |

| Table S3. $\boldsymbol{\chi}^{\boldsymbol{2}}$ tests of differences in perpetrator frequencies by age of onset | | | | |  |
| --- | --- | --- | --- | --- | --- |
|  | Neglect | Physical Abuse | Emotional Abuse | Sexual Abuse | Overall CM |
| Parents | $\chi^{2}$ (2)= 13.03,  p = .001** | $\chi^{2}$ (2)= 20.66,  p < .001** | $\chi^{2}$ (2)= 10.57,  p < .01** | $\chi^{2}$ (2)= 1.70,  p = .43 | $\chi^{2}$ (2)= 94.54, p < .001** |
| Siblings | $\chi^{2}$ (2)= 4.70,  p =.10 | $\chi^{2}$ (2)= 5.88,  p = .05 | $\chi^{2}$ (2)= 12.01,  p < .01 ** | $\chi^{2}$ (2)= 6.43,  p = .04 * | $\chi^{2}$ (2)= 26.74, p < .001* |
| Relatives and others in household | $\chi^{2}$ (2)= 8.30,  p = .02* | $\chi^{2}$ (2)= 9.83,  p < .01** | $\chi^{2}$ (2)= 6.01,  p = .05 | $\chi^{2}$ (2)= 29.28,  p < .001*** | $\chi^{2}$(2)=18.94,  p <.001** |
| Professionals in child-related environments | $\chi^{2}$ (2)= .82,  p = .67 | $\chi^{2}$ (2)= 7.94,  p = .02 *. | $\chi^{2}$ (2)= .09,  p = .96 | $\chi^{2}$ (2)= 3.74,  p = .15 | $\chi^{2}$(2)=3.64,  p =.16 |
| Other adults |  | $\chi^{2}$ (2)= 1.69,  p = .43 | $\chi^{2}$ (2)= 7.08,  p = .03*. | $\chi^{2}$ (2)=3.65,  p = .16 | $\chi^{2}$(2)=3.20,  p =.20 |
| Peers and partners |  | $\chi^{2}$ (2)=17.90,  p <.001** | $\chi^{2}$ (2)= .27,  p =.88 | $\chi^{2}$ (2)=11.51,  p < .01 ** | $\chi^{2}$(2)=7.21,  p =.03. |

**Figure S2.** Relative frequencies of perpetrators categorized by onset per subytpe

| **Table S4.** Partial correlations of CM multiplicity and Perpetrator relations | | | | | | | | |  |
| --- | --- | --- | --- | --- | --- | --- | --- | --- | --- |
| CM characteristic | 2 | 3 | 4 | 5 | 6 | 7 | 8 | 9 | |
| 1. Perpetrator Count | .85** | .74** | .74** | .63** | .67** | .60** | .50** | .43** | |
| 2.CM subtype multiplicity | 1 | .79** | .75** | .49** | .55** | .44** | .43** | .38** | |
| 3.Duration |  | 1 | .69** | .42** | .49** | .38** | .34** | .29** | |
| 4.Parents |  |  | 1 | .40** | .40** | .28** | .21** | .12** | |
| 5.Siblings |  |  |  | 1 | .31** | .23** | .17** | .14** | |
| 6.Relatives and others in household |  |  |  |  | 1 | .27** | .31** | .10** | |
| 7.Professionals in child-related environments |  |  |  |  |  | 1 | .26** | .17** | |
| 8.Other adults |  |  |  |  |  |  | 1 | .25** | |
| 9.Peers and partners |  |  |  |  |  |  |  | 1 | |
| *Note.* Controlled for age, sex, household net income, ** p <.001. | | | | | | | | | |
